# Supplementary material for: Sexual norms and the intention to use healthcare services related to female genital cutting: A qualitative study among Somali and Sudanese women in Norway
Source: PLoS One. 2020 May 18;15(5):e0233440. doi: 10.1371/journal.pone.0233440 (PMC7233551; doi:10.1371/journal.pone.0233440)
Supplement: S1 Appendix — (PDF) [file pone.0233440.s001.pdf]

Semi-structured interview guide

1. Background information
2. Knowledge, perceptions and expectations regarding female circumcision
    a. Prior to own circumcision
    b. After own circumcision – in country of origin/where circumcision took place
    c. After own circumcision – in Norway and/or other countries of migration
3. Own experience with female circumcision
    a. Preparation
    b. The actual circumcision
    c. Immediately after
4. Own experiences with perceived circumcision-related health problems (before migration)
    a. Immediately after.
    b. During childhood up to puberty.
    c. Puberty up to first sexual encounter/marriage.
    d. During marriage.
    e. Pregnancy and childbirth.
5. Own experiences with perceived circumcision-related health problems (after migration)
    a. During childhood up to puberty.
    b. Puberty up to first sexual encounter/marriage.
    c. First sexual encounter and afterwards.
    d. Pregnancy and childbirth.
6. Experiences of close friends and/or family members with perceived circumcision-related health problems
    a. Immediately after.
    b. During childhood up to puberty.
    c. Puberty up to first sexual encounter/marriage.
    d. First sexual encounter and afterwards.
    e. Pregnancy and childbirth.
7. How these health problems, if any, were explained or understood
    a. In countries of origin.
    b. In Norway.
8. How these health problems, if any, were dealt with (also ignored) and why.
    a. In countries of origin.
    b. In Norway.
9. Knowledge, perceptions and expectations regarding circumcision-related healthcare services
    a. In countries of origin.
    b. In Norway.
10. Experiences (positive and negative) with circumcision-related healthcare services
    a. In countries of origin.
    b. In Norway.
11. Unmet healthcare needs
12. Other?
