# Supplementary material for: Sexual norms and the intention to use healthcare services related to female genital cutting: A qualitative study among Somali and Sudanese women in Norway
Source: PLoS One. 2020 May 18;15(5):e0233440. doi: 10.1371/journal.pone.0233440 (PMC7233551; doi:10.1371/journal.pone.0233440)
Supplement: Attachment — Submitted filename: Response to Reviewers.docx [file pone.0233440.s002.docx]

07.02.2020

**Response to reviewers**

We would like to thank the academic editor and the reviewers for their valuable time and feedback.

In this letter, we provide point-by-point response to both the editor and reviewers.

# Response to the academic editor

1. Please ensure that your manuscript meets PLOS ONE's style requirements, including those for file naming. The PLOS ONE style templates can be found at

http://www.journals.plos.org/plosone/s/file?id=wjVg/PLOSOne_formatting_sample_main_body.pdf and <http://www.journals.plos.org/plosone/s/file?id=ba62/PLOSOne_formatting_sample_title_authors_affiliations.pdf>.

*Response: done.*

2. Please amend your current ethics statement to address the following concerns: Please explain why was written consent was not obtained, how you recorded/documented participant consent, and if the ethics committees/IRBs approved this consent procedure.

*Response: done. Also, amended in the methods section, Lines 189-196.*

3. You indicated that you had ethical approval for your study. In your Methods section, please ensure you have also stated whether you obtained consent from parents or guardians of the minors included in the study or whether the research ethics committee or IRB specifically waived the need for their consent.

*Response: In this study, we did neither obtain consent from parents or guardians of the minors (16-18 years old) included in the study, nor asked the ethical committee for a waiver of parental consent. According to the Norwegian Health Research Act (*[*https://lovdata.no/dokument/NL/lov/2008-06-20-44*](https://lovdata.no/dokument/NL/lov/2008-06-20-44)*), the general rule is that minors can give independent consent from the age of 16. The only exceptions to this rule are when minors in the age group 16-18 years are to participate in clinical drug trials, or when bodily interventions are to be performed. Parental consent is only required in connection with these two exceptions. This clarification is now added to the methods section lines 183-189.*

4. Please, include a copy of the interview guide used in the study, in both the original language and English, as Supporting Information, or include a citation if it has been published previously.

*Response: done. The original language for our semi-structured interview guide is English. This interview guide is now included as “S1 Appendix.* *Semi-structured interview guide”.*

# Response to reviewer #1

1. Thank you for the opportunity to review this paper. I really enjoyed reading it. The topic is of key importance for understanding migrant women’s views and experiences around FGC and related health service development. The paper is unusual in the richness of data and cultural understanding presented. I particularly liked the use of the theories (sexual scripts & theory of planned behaviour). The methodology and methods are well described and justified. I have no major comments on most sections of the paper except for the final ones (Discussion/Conclusion).
   a. Here, I feel it would be useful to highlight more explicitly what the implications are of the research for health service development and community engagement around FGC. For example, in the current Conclusion, there is a statement as follows: “This apparent unfulfilled need and other important insights provided in this article regarding subjective norms that could hinder/facilitate the utilization of FGC related healthcare services could prove valuable not only for Norwegian but also for other decision-makers and healthcare professionals during the planning and/or delivery of FGC-related healthcare services…..” I feel that the phrase “other important insights” warrants further elaboration. These are implicit in the Discussion but need to be made more explicit. I would suggest making your key insights clear (re-state them) and then link them to specific implications and recommendations.

*Response: done. Lines 692-698, and 700-704.Also in table 2.*

b. I also wonder whether you could comment on whether you think your research has deepened our understanding more generally of the meaning of FGC for migrant women – compared with the way it is represented in more traditional biomedically oriented health services research?

*Response: done. Lines 706-722.*

c. It would be nice to have a commentary and critique of how this work may help to frame western thinking in this area in a different way. And what are the implications of this for future research – and for prevention related efforts?

*Response: done. Lines 722-726.*

2. The paper is generally very well written. Does the abstract need sub-headings?

*Response: We have revised the abstract and used sub-headings. Lines 16-62.*

# Response to reviewer #2:

1. To increase intelligibility of the themes in the article, themes could be presented in a table

*Response: done. A summary description of the themes is now included as table 2. Lines 292-294.*

2. Should write/discuss validity and reliability in material and method.

*Response: In this article, we followed the consolidated criteria for reporting qualitative research (COREQ) checklist. We have also systematically addressed issues related to the quality of qualitative data (credibility, transferability, dependability and confirmability), which relatively corresponds to internal and external validity, and reliability. For example, in the methods and/or findings sections:*

- *To enhance dependability (reliability) and transferability (external validity), we provided comprehensive record and thick description of:*
  - *The theoretical framework, Lines 128-156.*
  - *Sampling, recruitment, and study participants, Lines 169-198.*
  - *Setting and data collection, Lines 202-231.*
  - *Data management and analysis, Lines 233-247.*
  - *Themes and sub-themes that we systematically illustrated by participants’ quotations, Lines 300-393, 394-455, 456-554, and 555-641.*
- *To enhance credibility (internal validity), we:*
  - *Interviewed the same participant more than once, thus allowing for member checking:*
    - *In second interviews, the first author discussed with each participants her interpretations and recollections of information given in the first interviews, probing for confirmations, corrections, and elaborations. Lines 220-223.*
    - *In the third interviews, the first author expanded upon and discussed individually with nine participants her preliminary interpretations of patterns and emerging themes. Lines 226-227.*
  - *Validated the findings in three group discussions with additional 17 Sudanese and Somali participants. Lines 229-231.*
  - *The first author repeatedly discussed codes and themes with the last author (REBJ). Hence, the ensuing interpretations and findings became a negotiated version of more than a one person’s frame of reference. Lines 237-238.*
- *To enhance confirmability, we provided detailed information on the personal characteristics of the interviewer (the first author) and her relationship with the participants under the sub-heading “reflexivity”. Lines 248-265.*

Yours sincerely

Mai Mahgoub Ziyada

MSc, MPhil and MBBS

PhD Candidate

Section for Trauma, catastrophes and forced migration - NKVTS

Institute of health and society, Faculty of Medicine - UiO

m.m.ziyada@nkvts.no
