# Supplementary material for: Sexual norms and the intention to use healthcare services related to female genital cutting: A qualitative study among Somali and Sudanese women in Norway
Source: PLoS One. 2020 May 18;15(5):e0233440. doi: 10.1371/journal.pone.0233440 (PMC7233551; doi:10.1371/journal.pone.0233440)
Supplement: Attachment — Submitted filename: Response to Reviewers.docx [file pone.0233440.s004.docx]

09.04.2020

**Response to reviewers**

We would like to thank the academic editor and the reviewers for their valuable time and feedback.

In this letter, we provide point-by-point response to the reviewers.

# Response to reviewer #3

1. Thank You for this opportunity to review this paper. I really find it interesting and informative about genital mutilation and prevailing social norms. You have really pick the important topic to discuss and present.

*Response: Thank you for your positive feedback. It is highly appreciated.*

2. Not much but few observation on the methodology section.
   a. Since you have mentioned this is qualitative study, it would be better if you mention what type of qualitative study it is e.g. explorative.

*Response: Done. Line 141.*

b. Also, regarding the age of respondent you have mentioned 16-63 years, i am not clear on what evidence you have choosen this age range. I personally feel that while taking any age range of respondent standard age range for the study will be considered appropriate

*Response: We agree that the standard age range for women of reproductive age-group (15-49 years) is the most appropriate age range for the study. As shown in table 1, all of our respondents, except for two key informants, are in the standard age-group. Based on the relevance of the individual experiences of these key informants to our research questions, we have decided to include them regardless of their age.*

c. The basis of choosing only 26 respondent.

*Response: We have stopped recruitment once no new themes were observed. This is now clarified in line 184.*

Additional comments from reviewer #3:

d. Arrange (the marital status in table 1) in ascending or descending order.

*Response: done.*

e. Is the guiding question same during each time of interview?

*Response: Yes, we used a semi-structured interview guide (see S1 Appendix).*

f. Why we interview same individual twice and thrice?

*Response: FGC is a personal and sensitive topic. Hence, our main aim during the first interviews was to build trust and invite the participants' own narrations of FGC related experiences (lines 190-191). In the second interviews we further encouraged the participants' own narrations of FGC related experiences by revisiting the narrations from the first interviews and probing for confirmations, corrections, and elaborations (lines 203-205). The main purpose of the third interviews was to member check our analysis by discussing our preliminary interpretations of patterns and emerging themes with the participants (lines 208-209).*

g. Why there is no recording of any interview? Isn't this will generate recall bias.

*Response: Only first interviews were not recorded. To minimize the risk of recall bias, MMZ recorded her recollections, as well as her interpretations of the interview immediately after each interview (lines 192-194).*

# Response to reviewer #4:

1. This is an excellent in-depth article on an important subject. The qualitative analysis is very well described and skillfully presented. The insights learned are valuable. My only suggestion would be to include a more specific description of the themes/findings in the results section of the abstract.

*Response: Thank you for your positive feedback, it is highly appreciated. A specific description of the themes is now included in the results section of the abstract (lines 31-34).*

Yours sincerely

Mai Mahgoub Ziyada

MSc, MPhil and MBBS

PhD Candidate

Section for Trauma, catastrophes and forced migration - NKVTS

Institute of health and society, Faculty of Medicine - UiO

m.m.ziyada@nkvts.no
